# The clinical effect of bone perforations in periodontal regeneration and alveolar socket preservation: a systematic review with meta-analysis

**DOI:** 10.1007/s00784-025-06152-4

**Published:** 2025-01-16

**Authors:** Paolo Pesce, Luigi Canullo, Tiziano Testori, Alessandro Mastroianni, Massimo Del Fabbro, Maria Menini

**Affiliations:** 1 Division of Prosthodontics and Implant Prosthodontics, Department of Surgical Sciences, University of Genova, Genova, Italy; 2 Dental Clinic, Section of Implant Dentistry and Oral Rehabilitation, IRCCS Galeazzi Sant’Ambrogio Hospital, Milan, Italy; 3 Department of Biomedical, Surgical and Dental Sciences, Università degli Studi di Milano, Via della Commenda 10, Milano, 20122 Italy; 4 Department of Periodontics and Oral Medicine, School of Dentistry, University of Michigan, Ann Arbor, Michigan USA; 5 Department of Oral Medicine, Infection and Immunity, Harvard School of Dental Medicine, Boston, MA USA; 6 University of Torvergata, Rome, Italy; 7 Fondazione IRCCS Ca’ Granda Ospedale Maggiore Policlinico, Milan, Italy

**Keywords:** Bone perforation, Bone regeneration, Decortication, GBR, Open flap debridement

## Abstract

**Objectives:**

The present systematic review aimed to evaluate if cortical bone perforation is effective in enhancing periodontal surgery and guided bone regeneration (GBR) in humans.

**Materials and methods:**

Electronic search was performed in PubMed, Scopus and Cochrane CENTRAL up to October 31st, 2023. Grey literature was also searched. Prospective controlled studies were included. Two PICO questions were created; one focusing on the effect of bone perforation in the treatment of peridodontal intrabony defects (primary outcome probing depth (PD)) and one focusing on the effect of bone perforation in guided bone regeneration (primary outcome: histologic and histomorphometric data). The risk of bias of the included studies was assessed using the Cochrane tool for randomized controlled studies (RCTs) and the Joanna Briggs Institute Critical Appraisal tool for cohort studies. Pairwise meta-analysis was undertaken when possible, to estimate the overall effect for the outcomes investigated.

**Results:**

The search on databases yielded a total of 653 articles. After screening, five RCTs and one non-randomized study were included. A meta-analysis was performed for the first PICO. PD was evaluated in 4 articles and no significant difference was found between the perforation vs. no perforation groups (0.11 mm (95% CI [-0.14 to 0.37 mm], *P* = 0.38). Additionally, radiographic defect depth (mean difference 0.77 mm, 95% CI [0.24 to 1.30 mm], *P* = 0.004) and distance between cemento-enamel junction and bone defect (standardized mean difference 0.98 mm, 95% CI [0.47 to 1.50 mm], *P* = 0.0002) resulted improved in the cortical bone perforation group.

**Conclusion:**

The evidence supporting a positive effect of using cortical perforations is very poor. Further studies with larger sample sizes are needed to determine whether decortication brings meaningful advantages.

**Clinical relevance:**

This study is focused on clinical studies and, using a rigorous study selection and a meta-analytic approach suggests that the apparent positive effect of bone decortication on the regeneration process still requires to be confirmed by more solid evidence.

## Introduction

Bone defects in the human jaw are a common occurrence, primarily attributed to factors such as premature tooth loss resulting from periodontal disease or trauma. Often, these conditions lead to a reduction in alveolar bone volume, which may jeopardize the rehabilitation through osseointegrated implants [1]. To facilitate implant placement in accordance with the prosthetically driven approach, bone regeneration or augmentation is frequently deemed necessary. This entails employing various materials and grafting techniques, which establish reliable procedures for endosseous implant placement [2–4]. The guided bone regeneration (GBR) technique was developed to simplify the placement of implants in partially or completely edentulous patients lacking adequate bone tissue [5, 6].

Similarly, for the treatment of periodontal intrabony defects, guided tissue regeneration (GTR) aims to restore lost periodontal structures and establish new connective tissues and alveolar bone support. In fact, clinical and histologic evidence showed that this procedure predictably leads to the healing of intrabony defects: GTR demonstrated a significantly better clinical attachment level (CAL) gain (on the average 1.15 mm) and probing depth (PD) reduction (1.24 mm) when compared to open flap debridement after 12-month follow-up [7]. Following GTR procedure, new cementum, periodontal ligament, and alveolar bone regeneration has been demonstrated in treated sites at six months post-op [8]. Although different procedures, GBR and GTR share the same biologic environment and therefore the same healing phases sharing the same biologic processes.

Healthy bones rely on robust blood circulation to deliver oxygen, essential nutrients, and remove metabolic waste products [9]. In the context of bone regeneration, angiogenesis assumes a pivotal role in establishing a functional connection between graft material and the surrounding environment. The presence of a mature and well-established vascular network can enhance and speed up regeneration process. Furthermore, the development of new bone and the resorption of graft material appear to be closely linked to angiogenesis [10, 11]. In the edentulous ridge, the vascular supply differs somewhat from that of the dentate ridge. In the tooth-supported ridge, the vascular supply comprises the supraperiosteal arteriolar complex, the capillary network within the gingiva and periodontal ligament, and the intraosseous arterioles. However, when teeth are lost, the blood supply via the periodontal ligament disappears, and the main source becomes the blood vessels on the periosteum [12]. To facilitate the access of progenitor cells and blood vessels to the graft area and promote angiogenesis, the superficial bone layer is often decorticated. This process (also named intramarrow penetration) consists of creating several one-mm-size or less small perforations in the cortical bone to expose the underlying spongy bone. The concept of bone desquamation, as described by Frost [13], involves a regionally accelerated process of increased bone healing in response to noxious stimuli. Other researchers have suggested that cortical perforation may enhance phase two wound healing, or osteogenesis, in which peptide factors like bone morphogenetic proteins are released locally, promoting the differentiation of mesenchymal cells into osteoblastic lineage [14]. The practice of perforating cortical bone before bone grafting is an integral component of the GBR procedure [15, 16]. Cortical bone acts as a physical barrier, impeding the migration of cells and tissues involved in bone formation. Perforating the cortical bone eliminates this barrier and promotes processes such as hemorrhage, progenitor cell migration, and angiogenesis within the graft area, contributing to expedited healing. Additionally, perforation can enhance the physical connection between the recipient site and the graft materials [15–17]. Such biological activities triggered by cortical perforations are expected to translate into improved clinical, radiographic and histological outcomes.

Past reviews on this topic concluded that the literature to fully support this concept is scarce, primarily due to the limited availability of human clinical studies and the variability in results from animal studies [18, 19]. In the recent years, however, the interest in bone decortication re-emerged and new scientific data were presented, so it seemed justified to analyze the current evidence through an updated review [20–22].

The aim of this systematic review was to determine the effect of cortical perforations in the tratmente of periodontal intrabony defects and alveolar ridge preservation.

## Methods

The present review was created following the PRISMA guidelines and the review protocol was registered on PROSPERO (submission No. CRD42023452758).

### PICO questions

Two focused questions were elaborated following the PICO format.

#### Bone perforations in periodontal surgery

The first PICO was: In patients undergoing periodontal surgery for intrabony defects, is the cortical bone perforation beneficial for reducing probing depth?

Population (P): All age participants that had one intrabony defect to be treated.

Intervention (I): Periodontal surgery with bone perforation.

Comparison (C): Periodontal surgery without bone perforation.

Outcome (O): primary outcome was probing depth reduction (PD); additional outcomes were clinical data: clinical attachment level (CAL), recession (REC), plaque index (PI), gingival index (GI) and radiographical data: intrabony defect depth, intrabony defect width, defect angle, distance between CEJ and bone defect.

#### Bone perforations in guided bone regeneration

The second PICO was: In patients needing guided bone regeneration, is the cortical bone perforation beneficial in terms of clinical and histological/ histomorphometric parameters?

Population (P): All age participants that had one bone defect to be treated through bone regeneration.

Intervention (I): Bone regeneration where the cortical bone has been perforated before the use of regenerative material.

Comparison (C): Bone regeneration where the cortical bone has NOT been perforated before the use of regenerative material.

Outcomes (O): primary outcomes were histologic and histomorphometric data; additional outcomes were clinical and radiographic parameters.

Electronic search was performed using three databases, PubMed (MEDLINE), Scopus and the Cochrane Central Register of Controlled Clinical Trials (CENTRAL). The last search was done on October 31st, 2023. The references list of the included studies and relevant systematic reviews were examined for additional eligible studies. No language nor date of publication restriction were applied.

In addition to the primary search in established databases, we extended our investigation to include grey literature sources, encompassing conference abstracts, proceedings, and theses. Our search specifically targeted databases such as www.opengrey.eu. The search strategy for each database is reported in the supplementary material.

Two authors (PP and LC) screened the titles and abstracts of all the retrieved records, to get a list of the eligible papers. Cohen’s Kappa statistic was employed to evaluate the inter-examiners agreement on the screening process. In instances of uncertainty, a third co-author (MDF) was consulted. The complete text of all eligible studies was retrieved and assessed by the same two authors (PP, LC), to make sure that inclusion criteria were met. Any doubts were discussed with a third co-author (MDF). For any excluded study the reasons for exclusion were documented.

### Eligibility criteria

This review included studies possessing the following criteria: (1) prospective comparative clinical trials and observational studies; (2) human studies involving subjects not affected by systemic diseases interfering with the healing process; (3) a minimum follow-up of at least three months for histologic and histomorphometric outcomes, and at least six months for clinical and radiographic outcomes. Conversely, articles were excluded based on the following criteria: (1) duplicate reports of prior trials; (2) unavailability of full texts; (3) case reports; (4) pilot studies; (5) animal studies; (6) in-vitro studies; (7) systematic reviews and meta-analyses.

### Data extraction

Following a meticulous study selection process, two authors (PP and LC) performed data extraction from included studies, utilizing a Microsoft Excel spreadsheet. The extracted information encompassed the year and journal of publication, authors, title, study design, surgical technique, biomaterial used if any, sample size, patient age, gender, follow-up period in months, study location, and the investigated outcomes.

### Outcome variables

The primary outcome was PD for the first PICO. Further outcomes were: probing depth (PD, mm), clinical attachment level (CAL, mm), recession (REC, mm), plaque index (PI), gingival index (GI), vertical distance between CEJ and bone defect (mm). Radiographic outcomes were: defect depth (mm), defect width (mm), defect angle (degrees),

The primary outcome was the percentage (%) of new bone formation, determined by histomorphometric analysis for the second PICO. Further outcomes were: % of residual graft, % of mineralized tissue, % of soft tissue, microvessel density (number of vessels/mm^2^).

### Risk of bias assessment

Two co-authors (MDF, PP) independently evaluated the studies for risk of bias. Randomized controlled trials (RCTs) underwent assessment using the Cochrane risk-of-bias tool, scrutinizing seven distinct bias categories (random sequence generation, allocation concealment, blinding of participants and personnel, blinding of outcome assessment, incomplete outcome data, selective reporting, other bias). For “Other bias” it was considered if a sample size calculation was performed, and if between-group homogeneity in the main outcomes at baseline was evaluated. Each item was scored as high risk, low risk or uncertain. A conclusive bias judgment was then assigned to each included article. RCTs were categorized as having a low risk if all items were judged at low risk, moderate risk if there was some item at uncertain risk but none at high risk, or high risk if at least one item was judged at high risk. Cohort studies underwent risk of bias assessment utilizing the Joanna Briggs Institute (JBI) Critical Appraisal Checklist for Cohort Studies, covering eleven different domains ^19^. The potential risk levels were classified as low, moderate, serious, or critical. Cohort studies were judged at critical risk of bias if one or more items were critical, at serious risk if more than 4 items were unclear, at moderate risk if 2 to 4 items were unclear, and at low risk if there was no more than one item unclear. In case of doubts or discrepancies, a third reviewer was consulted (LC) [23].

### Data analysis

Pairwise meta-analysis was undertaken when possible, to estimate the overall effect for the outcomes investigated. The intervention’s effect was represented as mean differences (MDs), accompanied by 95% confidence intervals (CIs). Standardized mean difference was used when a single study weight resulted greater than 80%. Heterogeneity among the included studies was evaluated using Cochran’s test for heterogeneity, with a significance threshold set at *P* < 0.1.

Meta-analysis was conducted using Review Manager (RevMan Version 5.4.1, The Cochrane Collaboration, 2020), and a random or fixed-effects model was utilized, as appropriate. When significant heterogeneity in protocols, patients characteristics and study design was detected, a random-effects model was applied. If heterogeneity persisted, studies with a high risk of bias were excluded, and the analysis was rerun. For studies evaluating quantitative periodontal parameters, the mean changes with standard deviations between baseline and the longest follow-up in the two groups was considered. To address missing standard deviations, methods outlined in Sect. 7.7.3 of the Cochrane Handbook for Systematic Reviews of Interventions, Version 5.1.0 [24], were employed, when feasible. A p-value of 0.05 was considered as the significance threshold.

## Results

### Study selection

The exploration of online databases, including MEDLINE (*n* = 406), CENTRAL (*n* = 114), and SCOPUS (*n* = 815), resulted in the identification of 1335 relevant articles. One doctoral thesis was identified through the grey literature research. No articles were added reading the references of included studies. Following the removal of duplicates, 653 articles underwent evaluation. Among these, 645 were excluded during the title or abstract screening phase as they did not meet the inclusion criteria. The remaining eight articles were subjected to full-text reading, leading to the exclusion of two additional papers. The kappa value for inter-reviewer agreement in the screening step was 0.95, indicating very good agreement.

**Fig. 1:**
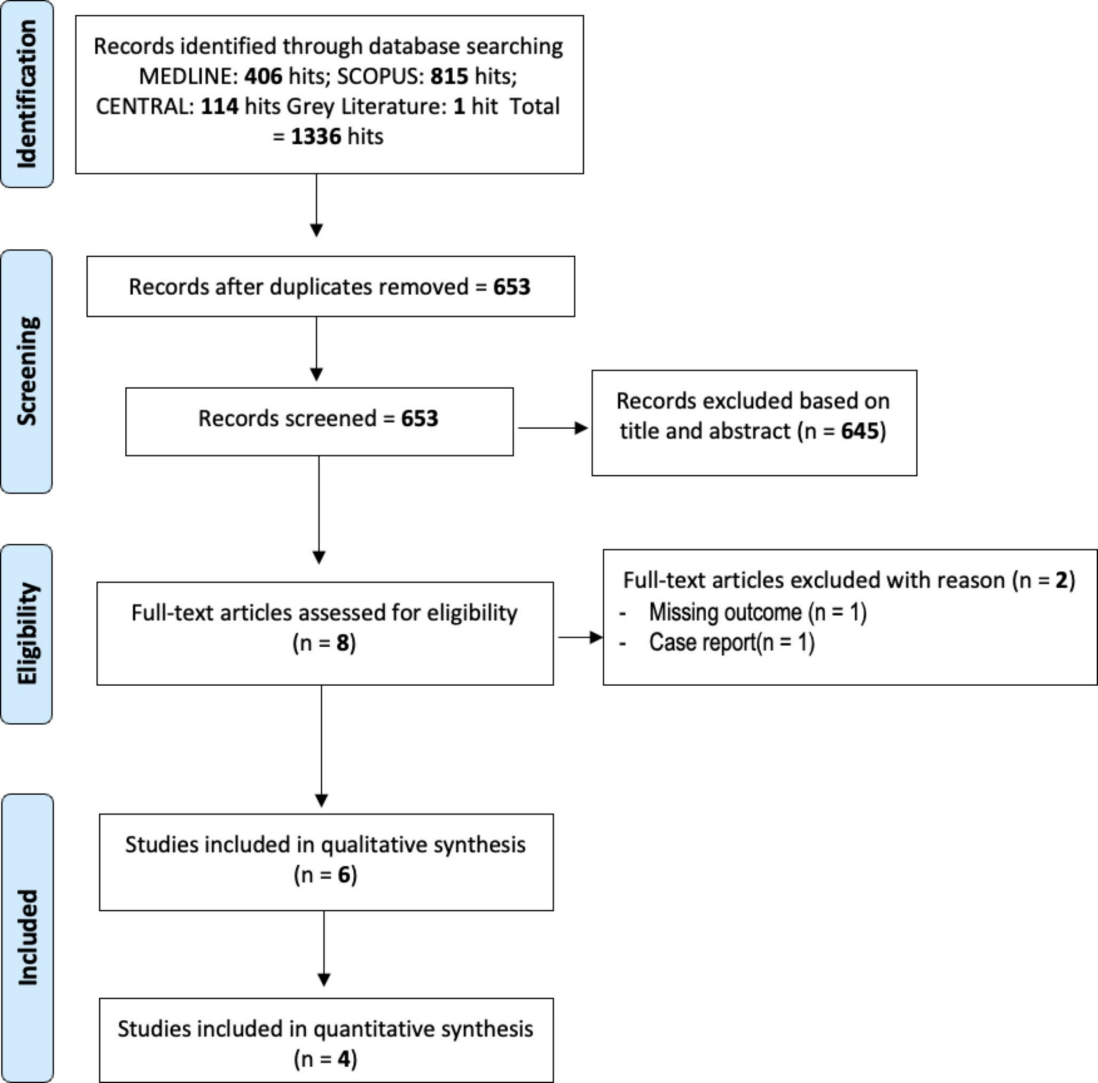
Flow chart of included studies

A total of six studies were ultimately included in the meta-analysis, with two focusing on alveolar ridge preservation [25, 26], and four on the treatment of intrabony defects [27–29]. The selection process is shown in Fig. 1.

### Description of included studies

Of the six articles included in the meta-analysis, five were randomized clinical trials [26–30], and one was a prospective clinical trial [25]. The main features of the included studies are reported in Table 1.

**Table 1:** Main characteristics of included studies

Authors Year	Study Design	Technique	Material	sample size (included)	sample size (FINAL)	Sex male (M)/ female (F)	Age Control, years, mean ± SD (range)	Age Test mean ± SD (range)	position	Follow-up	outcomes
Crea et al. [27]	RCT	open flap debridement with or without perforation	no graft	42 (28T/14 C)	41 (28T/13 C)	20 M / 21 F	53.2 ± 6.0 (43–60)	51.8 ± 7.6 (36–65)	5 maxilla C, 8 mandible C, 13 maxilla T, 15 mandible T	12 months	PD, CAL, REC, BOP, PI, keratinized tissue, radiographic defect depth (rDD), radiographic defect width (rDW), radiographic defect angle (ANG)
Saini et al. [28]	RCT	open flap debridement and DFDBA with or without perforation	DFDBA	40 (20T/20 C)	32 (16T/16 C)	16 M/ 16 F	37.19 ± 9.52 (29–56)	37.25 ± 6.92 (29–55)	10 maxilla C, 6 mandible C, 11 maxilla T, 5 mandible T	6 and 9 months	PI, GI, PPD, CAL, REC, BOP, radiographic defect width (rDW) and depht, area, bone fill, radiographic defect angle (ANG)
Sharma et al. [29]	RCT	open flap debridement, and A-PRF with or without perforation	no graft	20 (10T/10 C)	20 (10T/10 C)	NR	(22–60)	(22–60)	NR	3 and 6 months	PI, GI, PPD, CAL Distance from the alveolar crest (AC) to BOD defect depth; Distance from CEJ to the base of the defect (BOD); Distance from CEJ to AC.
Arcara et al. [30]	RCT	open flap debridement with or without perforation	no graft	20 (9T/11 C)	20 (9T/11 C)	12 M / 8 F	53 ± 6,0 (43–60)	51,8‡7,6 (36–65)	7 maxilla C, 3 mandible C, 4 maxilla T, 6 mandible T	12 months	PI, PPD, CAL, REC, BOP, rDD (radiographic defect depth)
Danesh-Sani et al. [25]	CT	guided bone regeneration	collagen membrane + deproteinized bovine bone	18 (9T/9 C)	18 (9T/9 C)	8 M / 10 F	52 median (25–72)	52 median (25–72)	mandible	7 months	thickness of the bone trabeculae (mm), the percentages of residual graft particles, newly formed bone, and soft tissue components (i.e., bone marrow and/or connective tissue), microvessels density
Tresguerres et al. [26]	RCT	regeneration - bone blocks	allogenic block	26 (13T/13 C)	26 (13T/13 C)	7 M / 19 F	56.46 ± 12.05	56.46 ± 12.05	Maxilla	4 months	newly formed bone (NB), fibrous connective tissue (CT), allograft remnant (AR), percentage of mineralized bone, percentage of bone volume/tissue volume (BV/TV) and trabecular thickness, number, and separation, microvessel density

RCT: randomized controlled trial; DFDBA: demineralized freeze-dried bone allograft; A-PRF: advanced platelet-rich fibrin; T: test; C: control; SD: standard deviation; NA: not reported; PD: probing depth; PPD: periodontal probing depth; CAL: clinical attachment level; REC: recession; BOP: bleeding on probing; PI: plaque index; GI: gingival index; CEJ: cemento-enamel junction

Two articles analyzed the influence of bone perforation in alveolar ridge preservation [25, 26]. Tresguerres et al. in a RCT analyzed the role of cortical perforations in allogeneic block grafting for lateral augmentation in maxilla [26]. Danesh Sani et al. [25] in a controlled trial analyzed the influence of bone perforations in GBR surgeries.

Four articles analyzed the effect of bone decortication in intrabony defects [27–30]. Crea et al. in a RCT analyzed the effect of bone decortication performed after open flap debridement [27]. Arcara analyzed 20 patients with the same protocol used in Crea et al. [30]. Saini et al. in a RCT evaluated the effect of cortical perforation on the surgery of intrabony defects treated together with demineralized freeze-dried bone allograft [28]. Sharma et al. in a RCT evaluated the use of PRF and decortication in the treatment of intrabony defects [29].

### Risk of bias

**Fig. 2:**
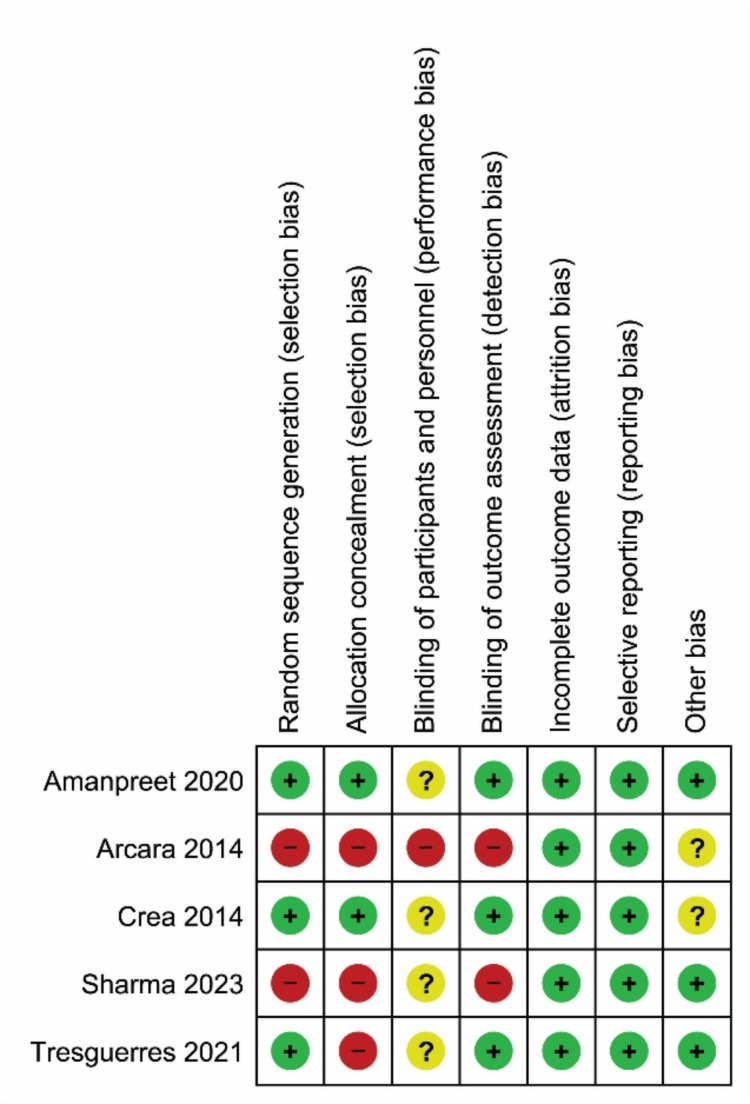
Risk of bias of included randomized studies

The risk of bias of the included RCTs is reported in Fig. 2. In our evaluation two of the included RCTs were classified at a moderate risk of bias [27, 28], and three at high risk [26, 29, 30] due to issues in the randomization process and in blinding procedures. The cohort study by Danesh-Sani et al. [25] was classified at moderate risk of bias because two items (n.5: “Were strategies to deal with confounding factors stated?” and n.11: “Was appropriate statistical analysis used?”) were judged as unclear, while one (n. 10: “Were strategies to address incomplete follow up utilized?”) was not applicable.

### Bone perforations in periodontal surgery

#### Probing depth, mm

**Fig. 3:**
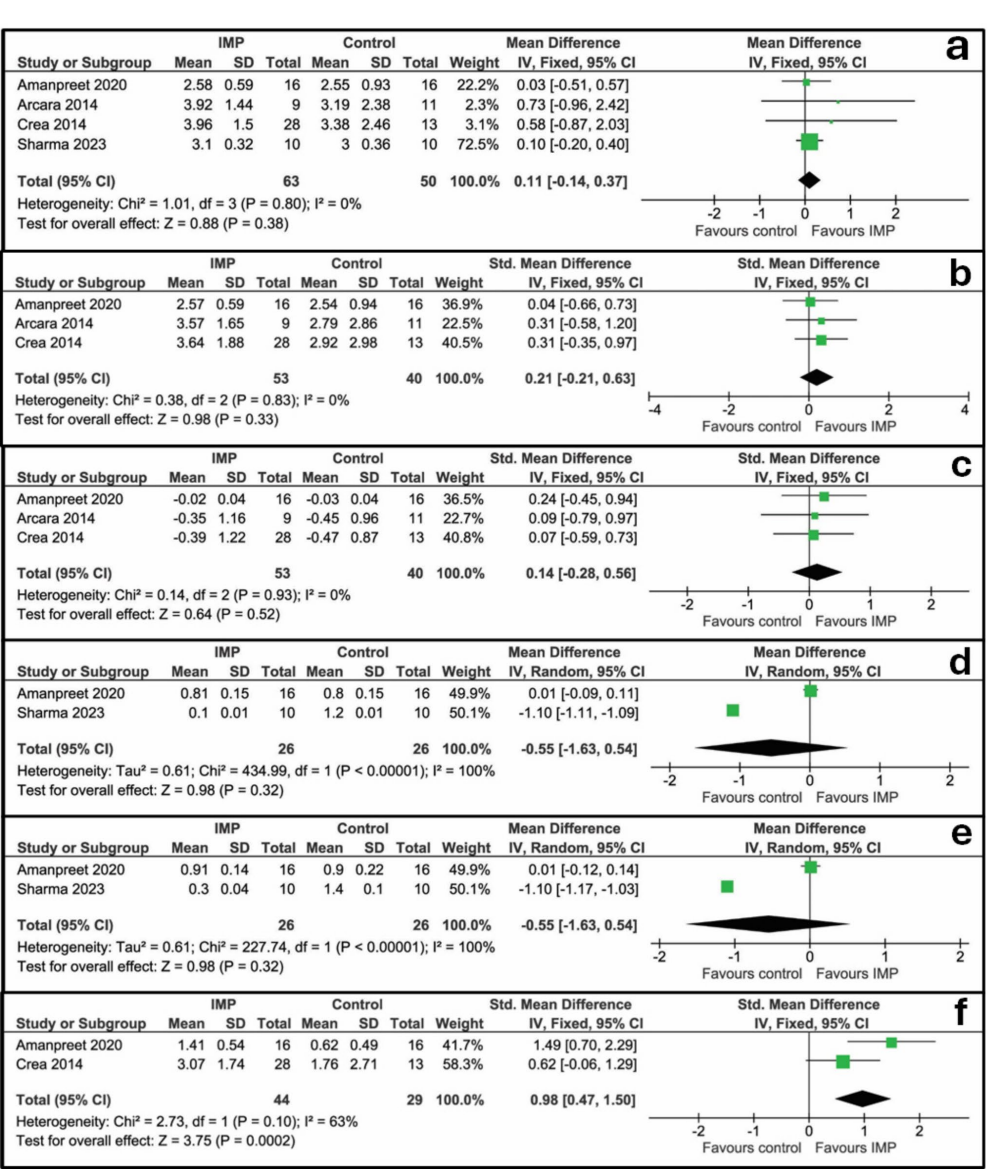
Forest plots of studies presenting clinical data. **a**: Probing Depth, mm; **b**: Clinical attachment level, mm; **c**: Recession, mm; **d**: Plaque Index; **e**: Gingival Index; **f**: Distance between CEJ and bone, mm

Probing depth was evaluated in four studies [27–30] and meta-analysis is reported in Fig. 3a. No significant difference was found between the perforation vs. no perforation groups (0.11 mm (95% CI [-0.14 to 0.37 mm], *P* = 0.38)). A very low heterogeneity among studies was found (I^2^ = 0%, *P* = 0.80).

#### Clinical attachment level, mm

Clinical attachment level was evaluated in three studies [27, 28, 30] and meta-analysis is reported in Fig. 3b. No significant difference was found between the perforation vs. no perforation groups (0.21 mm (95% CI [-0.21 to 0.63 mm], *P* = 0.33)). A very low heterogeneity among studies was found (I^2^ = 0%, *P* = 0.83).

#### Recession, mm

Recession was evaluated in three studies [27, 28, 30] and meta-analysis is reported in Fig. 3c. No significant difference was found between the perforation vs. no perforation groups (0.14 mm (95% CI [-0.28 to 0.56 mm], *P* = 0.52)). A very low heterogeneity among studies was found (I^2^ = 0%, *P* = 0.93).

#### Plaque index

**Fig. 4:**
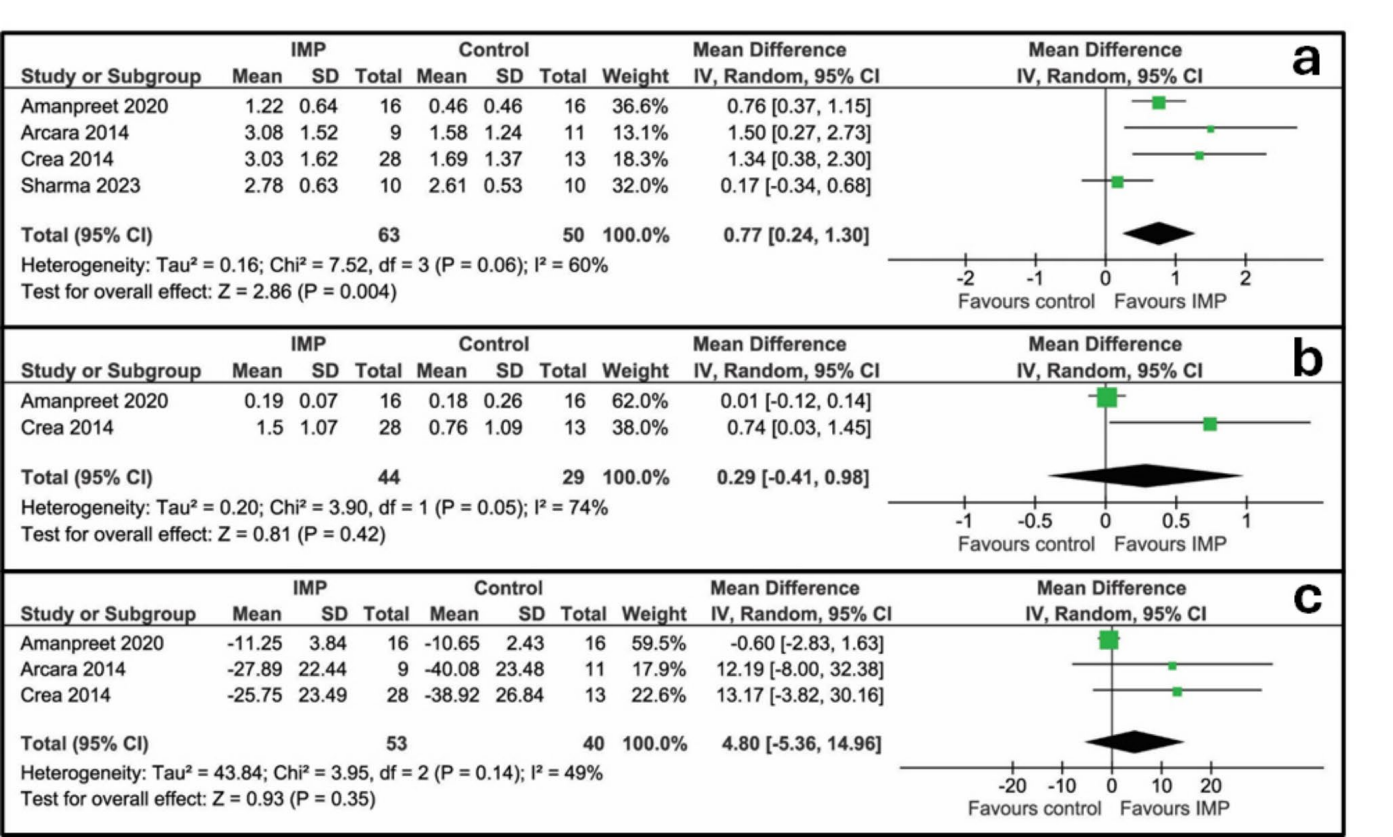
Forest plots of studies presenting radiographic data. **a**: defect depth, mm; **b**: defect width, mm; **c**: defect angle, degrees

Plaque index was evaluated in two studies [28, 29] and meta-analysis is reported in Fig. 4d. No significant difference was found between the perforation vs. no perforation groups (-0.55 (95% CI [-1.63 to 0.54], *P* = 0.32)). A high heterogeneity among studies was found (I^2^ = 100%, *P* < 0.00001).

#### Gingival index

Gingival index was evaluated in two studies [28, 29] and meta-analysis is reported in Fig. 3e. No significant difference was found between the perforation vs. no perforation groups (-0.55 (95% CI [-1.63 to 0.54], *P* = 0.32)). A high heterogeneity among studies was found (I^2^ = 100%, *P* < 0.00001).

#### Distance between CEJ and bone, mm

Distance between CEJ and bone was evaluated in two studies [27, 28] and meta-analysis is reported in Fig. 3f. Standardized mean difference was used because the weight of the study by Amanpreet K. Saini et al. ^24^ was greater than 80%. A significant advantage of the perforation over no perforation groups was found (0.98 mm (95% CI [0.47 to 1.50 mm], *P* = 0.0002)). A moderate heterogeneity among studies was found (I^2^ = 63%, *P* = 0.10).

#### Defect depth, mm

Defect depth was evaluated in four studies [27–30] and meta-analysis is reported in Fig. 4a. A significant difference was found between the perforation vs. no perforation groups (0.77 mm (95% CI [0.24 to 1.30 mm], *P* = 0.004)). A moderate heterogeneity among studies was found (I^2^ = 60%, *P* = 0.06).

#### Defect width, mm

Defect width was evaluated in two studies [27, 28] and meta-analysis is reported in Fig. 4b. No significant difference was found between the perforation vs. no perforation groups (0.29 mm (95% CI [-0.41 to 0.98 mm], *P* = 0.42)). A significant heterogeneity among studies was found (I^2^ = 74%, *P* = 0.05).

#### Defect angle, degrees

Defect angle was evaluated in three studies [27, 28, 30] and meta-analysis is reported in Fig. 4c. No significant difference was found between the perforation vs. no perforation groups (4.80 degrees (95% CI [-5.36 to 14.96 degrees], *P* = 0.35)). A moderate heterogeneity among studies was found (I^2^ = 49%, *P* = 0.14).

### Bone perforations in guided bone regeneration

Primary outcomes were reported in two studies, one RCT^26^ and one non-randomized prospective controlled study^25^. Because of the different study design, a meta-analysis was not undertaken, to avoid the risk of providing misleading results, and the study findings are summarized in Table 2.

**Table 2:** Histomorphometric findings

Study	Danesh-Sani et al. [25]		Tresguerres et al. [26]	
Outcome variable	Cortical perforation (*n* = 9)	Control group (*n* = 9)	Cortical perforation (*n* = 13)	Control group (*n* = 13)
New bone formation %	27.77 ± 11.32	25.33 ± 11.50	25.7 ± 11.2	22.3 ± 9.7
P-value	0.13		0.63	
Residual graft, %	34.78 ± 16.24	27.67 ± 10.0	39.3 ± 20.4	41.2 ± 22.7
P-value	0.09		0.72	
Mineralized tissue, %	Not reported		57.21 ± 10.6	53.9 ± 8.8
P-value			0.24	
Soft tissue, %	37.44 ± 14.93	47.0 ± 15.0	33.0 ± 14.7	36.5 ± 15.7
P-value	0.24		0.82	
Microvessel density, number of vessels/mm^3^	10.11 ± 2.86	5.44 ± 3.54	39.21 ± 10.53	34.16 ± 12.67
P-value	0.01		0.40	

#### New bone formation, %

Both studies found that the amount of newly formed bone was greater in the group using cortical perforations, but not significantly.

#### Residual graft, %

In both studies no significant difference was found between the cortical perforation vs. no perforation groups.

#### Mineralized tissue, %

This was only reported by Tresguerres et al. [26]. No significant difference was found between the group using perforation and the control group (*P* = 0.24).

#### Soft tissue, %

In both studies soft tissue proportion resulted greater in the control group, but without achieving significance.

#### Microvessel density, N. of microvessels/mm^3^

The mean number of microvessels per cubic mm resulted significantly higher in the perforation group than in the control group only in the study by Danesh Sani et al. [25] (*P* = 0.01), while it did not achieve significance in the Tresguerres et al. study [26] (*P* = 0.40).

## Discussion

Results of the present systematic review do not support a consistently positive effect of decortication in GBR. The primary outcome, as well as most of the parameters investigated, did not show clinically relevant improvements in the groups treated with decortication respect to control groups. Only in one randomized study cortical perforations were found to promote angiogenesis in alveolar preservation procedures ^25^. A meta-analysis of four RCTs on periodontal surgery found that decortication may reduce radiographic defect depth, as compared to control (Fig. 4a). Decortication of the recipient bone has been done in numerous clinical investigations as part of a guided bone regeneration regimen, with positive outcomes [31, 32]. On the other hand, some research on animals has shown that bone growth can happen even in the absence of decortication [33, 34]. Thus, it is still up for debate whether the recipient bone’s holes promote bone regeneration. Angiogenesis is a multi-step process that is thought to be essential before bone production. In order to nourish the transplanted site with osteo-progenitor cells and supply the necessary elements for bone development, new blood vessels must be present [18]. Angiogenesis typically starts with the blood vessels that are already there and may become exposed to the grafted site during flap reflection. This causes damage to the arteries that extend past the flap and into the surface of the bone [35]. It might only take a small amount of vasculature to tear in order to start the biological process of bone rebuilding [36]. A number of theories could account for the advantageous effect of bone decortication on bone formation, such as enhanced angiogenesis; prior research has demonstrated that an aperture through the medullary bone promotes angiogenesis and helps new blood vessels sprout into the regenerated bone [18, 36]. The recipient bone’s decortication creates a channel to a cancellous bone that is rich in vessels and supplies blood to the grafted area. Progenitor cells and cytokines proliferate in the region as a result [18]. Normal bone healing is facilitated by cortical bone penetration, which is thought to be a noxious stimulus that starts the regional acceleratory phenomena [37, 38]. Angiogenesis and the blood supply are significant factors in GBR [39]. The size of the perforations produced in the recipient bone is another aspect that could affect the quality of the regenerated bone. When Nishimura et al. compared the two perforation sizes in terms of how much bone was formed, they discovered that the bigger perforation size was linked to more rapid and substantial bone production during the early stages of healing [39].

Angiogenesis, which involves the creation of new blood vessels from an existing vascular network found in nearby soft and supraperiosteal tissues, provides this blood supply [12, 40, 41]. Research has demonstrated the indisputable role that the angiogenesis process plays in the development of blood vessels during regenerative processes like dentin-pulp complex and dental pulp regeneration [42]. Angiogenesis is essential to bone regenerations because it establishes a functional link between the host tissues surrounding the grafting material and the surrounding bone. Vascular networks that have developed and matured can support and quicken the regenerative processes. Decorticating the surrounding bone is recommended to help connect the blood vessels in the adjacent bone’s marrow and bone substitute materials, thereby promoting angiogenesis events [12].

In order for bone tissue to properly support, sustain, and protect the internal organs as well as maintain blood calcium homeostasis, such tissue is constantly changing. The main supporting component for teeth, the alveolar bone, is particularly susceptible to rapid and constant remodeling brought on by positional changes, tooth eruption, and the functional requirements of mastication [43, 44]. The remodeling cycle consists of osteoclasts’ resorption of the bone matrix and osteoblasts’ subsequent creation and mineralization of a new matrix. Intercellular communication is essential for both proper functional activity and complete cell differentiation between bone-forming and bone-resorbing cells. Through the production of cytokines that stimulate the progenitors of osteoclasts, osteoblasts regulate the breakdown of bone [45–47]. To dissolve the mineral and break down the organic matrix, osteoclasts release acids and proteases, which releases the growth factors that have been stored. The differentiation and functional activity of cells belonging to the osteoblastic lineage are regulated by bone growth factors, which include platelet-derived growth factor (PDGF), fibroblast growth factors (FGFs), transforming growth factor-β (TGF-β), insulin-like growth factors (IGFs), vascular endothelial growth factor (VEGF), and bone morphogenetic proteins (BMPs) [48]. In addition to vascular neoformation, bone tissue has the known ability to regenerate on its own. This process is similar to remodeling in that it involves a complex and multifaceted cascade of biological events (cell migration, proliferation, adhesion, and differentiation) that are controlled by various growth factors secreted by both reactive cells in the damaged site and by bone cells themselves [48–50].

Danesh-Sani et al. [25] and Tresguerres et al. [26] evaluated how perforations of the cortical bone before GBR maintain a good percentage of residual graft and increase the percentage of mineralized tissue. Unfortunately, they didn’t report statistically significant findings. The skeleton’s mineralized “hard” tissues have special biomechanical qualities that allow them to support the weight and motion of the body while serving as a supply of vital minerals for vital bodily processes. The discovery of calcium phosphate minerals in bone was initially documented by Scheele in 1771. Subsequently, X-ray diffraction pattern analysis and chemical composition analyses revealed that bone mineral was a form of hydroxyapatite [(Ca)_10_(PO_4_)_6_(OH)_2_], similar to geological apatite (a group of phosphate minerals). Subsequent research, however, showed that the minerals in bone are not uniformly composed [51]. It is now recognized that the matured bone mineral is a substituted crystalline phase of calcium phosphate, known as carbonated hydroxyapatite [52], despite some disagreements regarding the initial phase of the deposited mineral and its time-dependent transition to apatite. Bone crystals were accurately measured using atomic force microscopy, 3D stereoscopic TEM, and high-resolution transmission electron microscopy (TEM) on organic matrix-free bone samples. These investigations have unequivocally demonstrated that bone crystals are long, thin, nanoscale platelets [53].

According to Danesh-Sani et al. [25] and Tresguerres et al. [26] perforations of the bone cortex before GBR maintain unchanged or even decrease the amount of soft tissue around the bone graft. This statement, however, was not supported by their results. Typically, the oral mucosa consists of a coral pink masticatory mucosa and a shiny red alveolar mucosa [54]. The thin layer of nonattached alveolar mucosa is primarily made up of collagen fibers that are only loosely connected. The attached mucosa, on the other hand, is thick, keratinized, and made up of dense, well-organized collagen fibers. The masticatory mucosa is resistant to thermal, chemical, and physical insults because it is densely packed, stippled, and firm against the periosteum [55, 56]. Maintenance of teeth, periodontal ligaments, and dental implants depends on a sufficient quantity of attached keratinized tissue [57]. Furthermore, sufficient zones of attached keratinized tissue are needed for removable prosthetic devices in order to generate a vacuum between the mucosa and the denture base, which facilitates appropriate retention [58]. Nevertheless, some patients frequently need concurrent soft tissue reconstruction because they do not have enough soft tissue in their oral cavity, primarily as a result of gingival recessions, infections, trauma, and tumors [59].

Lastly, there is another important clinical aspect to consider during regenerative procedures, when cortical perforations are performed, the subsequent bleeding, due to adhesive properties of the coagulum, helps maintaining the graft more stable and attached to the bony walls thus facilitating membranes fixation.

To the authors’ knowledge this is the first systematic review focusing on the role of human bone perforation in periodontal regeneration and bone augmentation procedures. Alvira-Gonzales et al. in a recent systematic review analyzed 16 studies (15 conducted on animals and one on human) concluding, similarly to our work, that the evidence is limited [19]. Kofina et al. [60] analyzed in a systematic review the role of bone perforation during root coverage procedures finding no evidence to suggest the use of perforation in these surgical situations.

This systematic review and meta-analysis, however, has some limitations. These are primarily due to the stringent eligibility criteria, which focused on prospective clinical trials and observational studies involving patients without systemic diseases and conditions that might compromise bone healing. This approach resulted in the inclusion of only six studies (two on alveolar ridge preservation and four on the treatment of intrabony defects). The initial intention, according to the focus question, was to systematically revise the effect of cortical perforations on guided bone regeneration procedures. However since no study dealing with bone augmentation was retrieved, we only focused on intrabony defects and alveolar ridge preservation. The limited number of studies included, while restrictive, allowed for a focused systematic review and meta-analysis evaluating clinically meaningful outcomes. Additionally no meta-analysis of studies on ridge preservation could be performed, due to the different design of the two included studies (one RCT and one controlled trial) and the consequent possible bias due to study design heterogeneity, in spite of the similarity between the protocols and findings of the two studies. Finally, the results emerging from meta-analyses of only two studies with a small sample size (such as the plots in Figs. 3d-f and 4b), should be taken very cautiously. However, as stated in some documents of the Cochrane Collaboration, two studies is a sufficient number to perform a meta-analysis, provided that those two studies can be meaningfully pooled and their results are sufficiently “similar” ^61^.

## Conclusions

Cortical perforations showed no consistent benefits for promoting bone regeneration in alveolar preservation procedures and periodontal surgery compared to controls without perforation. Very few of the parameters investigated resulted in clinically relevant benefits but the evidence level remains poor. Anyway, no negative effects of such procedure, nor peri- and postsurgical complications, have been reported. As increasing bleeding at the healing sites may increase the availability of oxygen and nutrients, as well as of blood cells and platelet factors essential for promoting the healing process, intramarrow perforations might be considered in the procedures for the reconstruction of bone defects. Further comparative studies, with larger sample size, consisting of both animal and clinical prospective research, is needed to better establish the effects of decortication on the tissues and cells involved in the healing process, especially in guided bone regeneration procedures.

## Data Availability

All data supporting the findings of this study are available within the paper.
